# Healthcare professional communication behaviours, skills, barriers, and enablers: Exploring the perspectives of people living with Inflammatory Bowel Disease

**DOI:** 10.1177/20551029241257782

**Published:** 2024-05-22

**Authors:** Rachel L Hawkins, Eleanor Bull

**Affiliations:** 1 Manchester Metropolitan University, UK; 2 7315 The University of Sheffield, UK; 3 5292 The University of Manchester, UK

**Keywords:** behavioural science, healthcare professionals, inflammatory bowel diseases, patient-provider communication, qualitative research

## Abstract

This qualitative study conceptualised effective communication behaviours of healthcare professionals (gastroenterologists, surgeons, nurses, and general practitioners) and explored communication barriers and facilitators from the perspective of adults with Inflammatory Bowel Disease (IBD). Seventeen qualitative interviews were conducted with people living with IBD in the UK or USA (*n* = 17) and their spouses (*n* = 4). An inductive content analysis was firstly applied to participants’ accounts to define which healthcare professionals’ behaviours and skills were perceived as essential for effective communication. An inductive reflexive thematic analysis elucidated themes of perceived barriers and facilitators experienced when communicating with their IBD healthcare professionals. Thirty-three provider communication behaviours were grouped into nine healthcare professional skills. Five themes encompassed 11 barriers and facilitators: professionals’ knowledge and behaviour, unequal power, patient navigation skills, time constraints and demand, and continuity and collaboration of care. For patients and some spouses, enhancing communication in IBD services means increasing patient, family, and health professional knowledge, encouraging collaborative partnership working, and promoting healthcare professional skills to communicate effectively within the reality of time restraints.

## Introduction

Crohn’s Disease (CD) and Ulcerative Colitis (UC), common forms of Inflammatory Bowel Diseases (IBD), are lifelong inflammatory conditions of the gastrointestinal tract characterised by a relapsing and remitting pattern of bowel and other symptoms (Tontini et al., 2015). IBD affects nearly seven million people globally with a particular burden in the Western World (Alatab et al., 2020). In the United Kingdom (UK), nearly half a million people are living with IBD, with this number expected to be greater with many misdiagnosed or awaiting a diagnosis (IBD UK, 2021). A diagnosis of IBD commonly occurs between ages 15–35 years and common physical symptoms across the life course include diarrhoea and or incontinence, abdominal pain, chronic fatigue, weight loss and a weakened immune system (Norton et al., 2013). In addition to managing the unpredictable and potentially stigmatising symptoms, complications arising from IBD may include bowel strictures resulting in the need for emergency surgery (Norton et al., 2013).

Many individuals with IBD (referred to as patients in our study as a term still commonly used and preferred within healthcare systems) (Daniel et al., 2019) endure single or combined pharmacological interventions, often requiring emergency surgical interventions (O’Connor et al., 2013) and life-long interactions with primary and secondary care healthcare professionals (HCPs) (O’Connor et al., 2013; Lamb et al., 2019) to support the adjustment and self-management of their condition. Individuals with IBD will encounter many HCP interactions, including with gastroenterologists, IBD surgeons, IBD nurses, general practitioners (GPs) and other allied health professionals, such as dieticians (Lamb et al., 2019). Patients living with IBD perceive family members, particularly spouses and partners to be integral to living well with IBD, including in making treatment decisions, and are commonly impacted by the disease (Golics et al., 2013). Research suggests that individuals with IBD commonly experience psychological and psychosocial difficulties in living with IBD (Häuser et al., 2014; Sun et al., 2019).

Effective communication between individuals and their HCPs is fundamental for positive health outcomes across various long-term conditions (Plevinsky et al., 2016; Vermeir et al., 2015). This is through, for example, supporting patient and family-centred care, informed decision making and helping people to adopt positive self-management health behaviours (Cheng et al., 2015; Windover et al., 2014). The patient and family-centred care approach recognises the importance of family across the individual’s illness trajectory with the potential to facilitate (or in some cases impede) positive health outcomes and health professional relationships (Clay and Parsh, 2016).

Current clinical guidelines in the UK advise IBD services to offer ‘clear and comprehensive communication’ (Kapasi et al., 2020) for people living with IBD. Effective communication with HCPs may promote perceived support and reduce the risk of psychological co-morbidities (Kourakos et al., 2017; Plevinsky et al., 2016). However, the majority of NHS patient complaints are thought to be secondary to a breakdown in communication between healthcare providers and patients (Abdelrahman and Abdelmageed, 2017) and negative patient experiences of healthcare communication are linked to risk of adverse events, discontinuity of care and inefficient use of resources (Vermeir et al., 2015). Patient-physician relationship has also been highlighted as crucial to informed decision making about IBD surgery (Lai et al., 2019). In the UK, the ‘making every contact count’ (MECC) initiative recognises the importance of healthcare professionals initiating conversations with patients around their health and wellbeing (Nelson et al., 2013; Public Health England, 2016).

Most models of adjustment to chronic conditions and behaviour change tend to focus on individual cognitions and behaviours rather than dyad communication but have many implications for healthcare communication and relationships. For instance, Moss-Morris (2013) Unified Theory of Adjustment focuses on cognitions and behaviours of people living with chronic conditions (Moss-Morris, 2013). The challenge of managing relationships with health professionals is considered as a type of ‘ongoing illness stressor’ with the potential to disrupt emotional equilibrium. Additionally, coping by seeking social support and adherence to medical and self-management regimes are viewed as factors helpful for adjustment, suggesting that the quality of relationship with healthcare professionals is both essential for, and a sign of, positive adjustment. Models of behaviour change such as the Health Belief Model (Rosenstock, 1974) and Common-Sense Model of Self-Regulation (Leventhal et al., 2016) explore the importance of health-related beliefs such as those surrounding the identity, timeline and perceived control over illness, in influencing individuals’ coping responses. These imply that professionals must work to explore and understand an individual’s health beliefs to provide tailored knowledge and shape understanding. Self-Determination Theory (Deci and Ryan, 1985), further emphasises how communication to support people’s experience of autonomy, competence and relatedness can foster their intrinsic motivation, including towards self-management.

However, none of the above models specifically detail what helpful healthcare communication behaviours or skills would look like in practice. It may be that some aspects are more important than others for people with IBD. For instance, recent qualitative research with people with IBD highlighted pain and pain communication to be a significant part of their overall IBD experience (Sweeney et al., 2019). Recommendations gathered from experts by experience, could have important implications for improving quality of care and HCP-patient interactions in IBD healthcare and add an important dimension to triangulate the existing literature (Papageorgiou et al., 2023)

Existing research suggests dissatisfaction among people with IBD surrounding their interactions with healthcare professionals (Khan et al., 2016; Vegni et al., 2018). Studies highlight issues of insufficient information from HCPs and that the psychosocial, emotional and psychological impact of IBD remain under-addressed (Fourie et al., 2018; Khan et al., 2016; Plevinsky et al., 2016; Vegni et al., 2018). A previous systematic review of HCP-patient dialogue in IBD found that conversations tend to be disease-centred (medications, patient symptoms, disease progression) and without consideration of the interpersonal elements of living with IBD (Karimi et al., 2021). For changes to occur to improve healthcare quality and patient satisfaction, it is essential to better understand this issue from the perspectives of patients and also their families as crucial agents in the IBD journey.

‘Communication’ encompasses a range of human behaviours and skills in practice, and can refer to the use of verbal and written words, along with non-verbal cues (Webster, 2013). Existing models of patient-professional communication emphasise the dynamic process of integrative information gathering, and a focus social dynamic and the relationship, environmental factors, and mutual expectations (Cheng et al., 2015; Frederikson, 1993; Kurtz, 2003; Windover et al., 2014). It is not clear what behaviours and skills people with IBD feel are integral to effective health professional communication and what barriers and facilitators may be acting as important influences. This is needed in order to ensure target behaviours in best practice guidelines are clear, evidence-based, patient-centred and implementable (Michie and Johnston, 2004; Pagoto et al., 2007).

Qualitative methods in healthcare voice the patient-perspective of chronic illness and offer a rich, in-depth insight into health professional-patient interactions. As such, qualitative approaches are appropriate methods for identifying behaviours of communication and are recognised for their contribution to informing clinical practice (Braun and Clarke, 2019). This study reports the findings of a qualitative exploration of patient perspectives surrounding two related research questions:

1. What do patients with IBD perceive as effective communication practices which can help build the patient-professional relationship and facilitate their condition self-management?
2. What do IBD patients perceive as the barriers and facilitators to effective communication with health care professionals?

With the present shift towards patient- and family-centred care (Clay and Parsh, 2016) and shared decision-making within health care delivery, this study aimed to offer a psychological and behavioural approach to understanding communication between IBD patients and their HCPs, to inform evidence-based practice within IBD health services (Dombrowski et al., 2016; Fourie et al., 2018).

## Methods and methodology

### Design

This study utilised qualitative methods to address the research enquiry. This enabled the unfolding of a rich understanding of the perceptions and experiences of communication of patients (Braun and Clarke, 2014). Qualitative research methods recognise the socially constructed nature of communication with HCPs (O’Mahoney, 2016). The study is reported in line with the Consolidated Criteria for Reporting Qualitative Research (Tong et al., 2007), to ensure high quality reporting and replicability of methods, see Appendix A in supplemental material.

### Participants

Twenty-one participants were recruited through website and social media advertising by UK IBD charities. The UK charity, Crohn’s and Colitis UK advertised the study through their website and on the platform Twitter (now known as X), whilst X and Facebook advertisements were posted on ‘Guts UK’ and ‘For Crohn’s’ media platforms, as well as the researchers’ own platforms. Hashtags including *“#IBD” “#Experiences #TalkingWithHealthcareProfessionals #FamilyMembers”* were used to reach IBD national support groups and X users with IBD. Participants viewed the study advertisement and inclusion criteria and were recruited by volunteer sampling. Eligible participants to take part in this study were aged 18 and over, given that the study focussed on experiences in adult services and experiences in paediatric services may be very different. Participants were also required to have a diagnosis of CD or UC given the study’s focus, and be English-speaking, because the study was unfunded without access to interpreters. Recognising the importance of family-centred care and the sensitivity of asking people to talk about emotive aspects of their illness, we also included family members in the inclusion criteria. All participants were invited to ask a family member to participate in the study interview with them if this was preferred.

Recruitment continued until data saturation was achieved upon when the ability to obtain new information regarding the objectives of this research were obtained, and therefore further coding was no longer viable (Fusch and Ness, 2015). This was gathered by collating notes and reflections from each interview in a reflexive log, which was continually reviewed by the lead researcher. This reflexive process was continually reviewed by the researcher and information power was also reviewed when reflecting on saturation (Braun and Clarke, 2021). The researchers agreed that data saturation had likely been reached when no new codes were identified from two consecutive interviews.

### Data collection

Data were collected by remote semi-structured interviewing by one female researcher (RH, MSc) who was conducting a postgraduate degree in Health Psychology. Participants were informed that the researcher was conducting the study as part of her postgraduate degree. Interviews were performed over telephone, Skype, or Zoom, depending on participant preference. All participants had no prior relationship with the researcher. The researcher had a personal experience of IBD – and therefore kept a reflexivity log throughout the research process. Interview questions were guided from a literature review of the IBD and communication which identified gaps in knowledge. The interview schedule undertook numerous piloting sessions with an individual with IBD who did not participate in the study. All participants chose a pseudonym. Open-ended questioning enabled the retrieval of rich, inductive data from participants (Ogden and Cornwell, 2010). Participants responses to HCP communication behaviour were probed further to elicit meaning, and to understand the perceived barriers and facilitators related to that behaviour (Shenton, 2004).

### Data analysis

Both an inductive thematic content analysis (TCA), and inductive reflexive thematic analysis (TA) were applied to the data set in order to answer both research questions. TCA was first applied to identify from participants’ accounts which communication behaviours and skills of HCPs were perceived as central to effective communication (research question 1). Findings from this element of the analysis was taken in response to the question “what does good communication with a healthcare professional look like to you?”. The inductive TCA conceptualised these behaviours and skills by identifying dominant communication behaviours and skills by frequency counts and coded the behaviours discussed by participants (Vaismoradi and Snelgrove, 2019). An inductive approach enabled the category development of positive communication practices from the patient and family member perspective specific to the context of IBD healthcare delivery (Kondracki et al., 2002). Other qualitative studies have used this two-part approach to analysis, using a CA to understand prominent codes to understand one element of the research question along with a TA to understand themes across datasets (Ngoma and Adebisi, 2023).

Secondly, an inductive reflexive TA of the perceived barriers and facilitators to effective communication with HCPs was performed, from the other interview questions, to answer research question 2. TA involves identifying, categorising, describing and presenting themes within a qualitative data set (Braun and Clarke, 2006). This method provides a systematic, robust framework for coding and identifying patterns in the data relating to the research aims (Braun and Clarke, 2014). To ensure rigour and credibility within this study, the stages of the TA were guided by Braun & Clarke’s six stages (Braun and Clarke, 2006). A person-centred, inductive approach was applied that centred upon the experiences of the participants of this study which were used to generate codes and themes relating to the barriers and facilitators at the latent level.

### Process of analysis

Participant interviews were audio-recorded and manually transcribed. One researcher (RH) immersed themselves into the data, repeatedly reading the data to familiarise with the content, and enabling inductive category development to take place (Braun and Clarke, 2006; Hsieh and Shannon, 2005). Transcription and coding were performed concurrently with the data collection, increasing awareness and understanding for saturation. Transcripts were read line by line, and identifying statements related to communication behaviours, barriers and facilitators were coded. Please refer to Supplemental File one and two for the full list of initial codes. The coding and generation of themes was performed using NVivo software and by hand. NVivo produces frequency counts of codes, and systematically orders codes to be clearly interpreted.

For the inductive CA of HCP behaviours and skills, all the manuscripts were read line by line and behaviours and skills elicited as important for positive communication with HCPs from participants were coded. Skills and behaviours of communication perceived most important to participants were captured by the frequency across the participant interviews. Clustering of coded behaviours enabled the category development of clustered skills into themes. The second researcher (EB, PhD) reviewed behaviours clustered into HCP skills against the transcript data. Six behaviours and two themed HCP skills were relabelled by the second researcher (EB), through discussion and agreement with the first researcher to which there were no disagreements.

In the inductive TA, after initial coding of participant quotes, codes were clustered together to form themes that represented the barriers and facilitators discussed by participants. These were then grouped into overarching higher order themes. Quotes from the participant manuscripts were selected based on how illustrative they were of the key concepts of the theme and their representation of the patterns within each theme of the dataset (Lingard, 2019). Thematic maps and thematic tables were used to demonstrate coding and theme generation, thereby increasing transparency by illustrating the progression of themes to the readers (Vaismoradi et al., 2016). The coding was performed by the lead researcher (RH) and checked by a senior health psychology researcher (EB), with areas of disagreement discussed during online meetings and decisions reached by consensus.

### Ethical considerations

This study received ethical approval from Manchester Metropolitan University research ethics committee, and followed procedural and conceptual guidelines from the British Psychological Study (BPS) Code of Human Research Ethics (British Psychological Society, 2021)

## Results

### Sample

A total of 21 participants took part in this study. Seventeen remote semi-structured interviews were conducted and analysed (13 interviews with IBD patients, four joint interviews with patients and their spouses) via telephone (*n* = 9), Skype (*n* = 3) or Zoom (*n* = 5). Participants of this study included adults with a diagnosis of IBD (11 Crohn’s Disease, 5 Ulcerative Colitis, 1 Ulcerative Proctitis) and their spouses (*N* = 4) (Table 1).

**Table 1.** Outline of participant demographics.

Interview number. and type	Pseudonyms	Diagnosis	Gender	Ethnicity	Age	Time since diagnosis
1 Telephone	Lucie	Crohn’s disease	Female	White British	31	2 years
2 Zoom	Richard and Jo (spouse)	Ulcerative colitis	Male	White British	56	15 years
3 Telephone	Oddbods	Crohn’s disease	Male	White British	39	7 years
4 Telephone	Andrew	Crohn’s disease	Male	White British	62	30 years
5 Telephone	Natalie	Ulcerative colitis	Female	White British	37	19 years
6 Telephone	Louise	Ulcerative colitis	Female	White British	51	27 years
7 Telephone	Ann	Crohn’s disease	Female	White British	57	38 years
8 Telephone	Minnie	Ulcerative proctitis	Female	White British	35	4 years
9 Skype	Lisa	Crohn’s disease	Female	White British	33	2 years
10 Zoom	Hector	Crohn’s disease	Male	White British	57	27 years
11 Zoom	Saul	Ulcerative colitis	Male	White British	27	13 years
12 Zoom	Elizabeth	Crohn’s disease	Female	White American	40	2 years
13 Zoom	Sophie	Ulcerative colitis	Female	White British	48	13 years
14Telephone	Roy	Crohn’s disease	Male	White British	45	29 years
15 Telephone	James and Sarah (spouse)	Crohn’s disease	Male	White British	50	28 years
16 Skype	Jane and Michael (spouse)	Crohn’s disease	Female	White British	47	5 years
17 Skype	Marcia and David (spouse)	Crohn’s disease	Female	White British	61	46 years

During the interviews, participants discussed their experiences of their interactions with primary and secondary HCPs. Seven participants had encounters with both public and private IBD health services. Interactions with personnel from various healthcare disciplines were discussed. The HCPs discussed included GPs (GPs), IBD nurses, IBD specialist consultants and gastroenterologists, surgeons, and dieticians.

### Healthcare professional communication behaviours and skills

The TCA identified key communication practices of HCPs perceived by participants as fundamental for effective communication between individuals with IBD, spouses and HCPs during consultations. In total, thirty-three health professional communication behaviours were identified that were then grouped into eight overarching themes, which in this case were labelled as healthcare professional ‘skills’. The eight skills included: Active and responsive listening skills, promote patient autonomy, provide timely outpatient care, offer personalised care, provide helpful self-management information, tailoring language when providing information, multidisciplinary communication, and body language and social cues during consultations. These themes represented the nuanced behaviours of good communication identified by participants. Table 2 displays these themed skills as a communication checklist.

**Table 2.** Healthcare professional communication behaviours and skills checklist.

Healthcare professional skill (theme)	Coding frequency	Behaviours
Active and responsive listening skills	5	Using open-ended questions
	7	Enabling questions from patients and spouses
	2	Paraphrasing information
	5	Listen to the patient/family members views
	4	Consider information provided by the patient/family member
	1	Establish consultation expectations of the patient
Promote patient autonomy	3	Allow patient to make their own choices
	2	Encourage patient to explore the different options for biologics
	1	Ask the patient to track their own progress
Provide timely outpatient care	4	Provide regular check-up opportunities
	2	Timely follow-ups during a flare
	1	Timely referrals from GPs
	2	Efficient at sending forms for tests
Offer personalised care	4	Ask the patient what is worrying them
	2	Ask about family history or comorbidities
	5	Show interest in the person’s life beyond their illness
	10	Ask the patient about their psychological wellbeing
Provide helpful self-management information	5	Discuss the risks and benefits of procedures/medication
	4	Give detailed information about medication
	4	Provide facts to back up information/research evidence
	4	Provide scenarios
	3	Use visuals e.g., handouts
	3	Check the patient understands the information
Tailoring language when providing information	1	Signpost to support services
	1	Repeat information if needed
	2	Use medical terminology
	1	Use layman’s terms
Multidisciplinary communication skills	11	Communicate with other primary and secondary care professionals involved in direct care (GP, gastroenterologist, IBD nurses, surgeons, dieticians)
	3	Communicate with GP colleagues when ordering investigations or prescribing new treatments
Body language and social cues during consultations	6	Use eye contact with the patient
	1	Respond empathetically when distressed
	3	Face the patient/family member during consultations
	1	Turn computers/charts so patients can see information

Participants of this study also detailed the context of when and with whom certain behaviours and skills during their interactions with HCPs were important to facilitating communication. Depending upon the patients’ stage of illness trajectory (newly diagnosed vs living with IBD >5 years), specific behaviours of HCPs were perceived as important for communication that facilitated decision-making and condition management. For example, behaviours such as enabling patient and spouse questioning, signposting to support services, tailoring language, and checking understanding of information was particularly important to participants during the early stages of their IBD diagnosis. This was discussed by participants both newer to living with IBD and with those who lived with IBD for over 10 years at the time of the research interview. These behaviours and skills were also important to participants when making informed decisions with their HCPs regarding pharmacological interventions, which occurred across the stages of illness trajectory.

In addition to the stage of illness trajectory, emphasis of behaviours was also placed upon GPs in primary care services. Multidisciplinary communication skills, and active and responsive listening skills were emphasised, particularly by people living with IBD in this study, who often felt these behaviours were lacking amongst GPs. Following referral from a GP to IBD specialist clinics, providing information and body language and social cues during consultations were nuanced by the participants as important behaviours of their consultants during consultations.

### Barriers and facilitators

**Figure 1.**
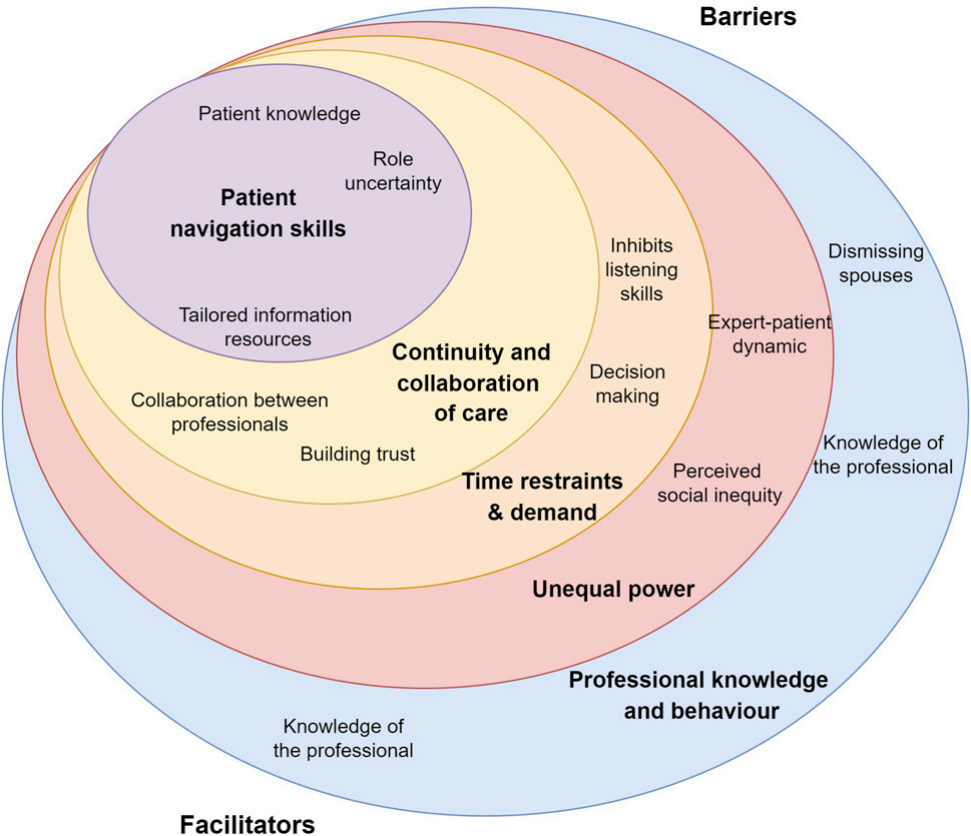
Diagram eliciting themes and subthemes relating to barriers and facilitators of effective communication between IBD patients and their healthcare professionals.

Following an inductive TA, five overarching themes incorporating 11 barriers and facilitators to effective communication were identified. The five themes were: HCP knowledge and behaviour, unequal power, patient navigation skills, time constraints and demand, and continuity and collaboration of care (Table 3 and Figure 1).

**Table 3.** Summary of themes.

Overarching Theme	Subthemes	Barrier/Facilitator
Professional knowledge and behaviour	Knowledge of the professional	Barrier and facilitator
	Dismissing spouses	Barrier
Unequal power	Expert-patient dynamic	Barrier
	Impact of perceived social inequity	Barrier
Patient navigation skills	Patient knowledge	Barrier
	Role uncertainty	Barrier
	Tailored information resources	Facilitator
Time restraints and demand	Inhibiting listening skills	Barrier
	Decision-making	Barrier
Continuity and collaboration of care	Building trust	Facilitator
	Collaboration between professionals	Facilitator

### Theme 1: Healthcare professional knowledge and behaviour

Both barriers and facilitators to communication were conceptualised as relating to factors driven by health professional knowledge, attitudes, and behaviour. This theme is broken down into subthemes of ‘knowledge of the professional’, and a barrier of ‘dismissing spouses.’

#### Subtheme: Perceived knowledge of the professional

Perceived knowledge of the HCP emerged as both a barrier and facilitator amongst 14 participants. In this instance, HCP ‘knowledge’ related to the diagnosis, treatment and management of IBD. One participant elucidated the importance of knowledge of their IBD nurses that facilitated explanation to patient questioning:

They’re so knowledgeable…they explain things more, and more tests and what the medication does. They know so much and they’re just more prepared to answer questions (Louise, aged 51, CD)

Louise’s confidence in her nurses’ knowledge facilitates communication by promoting information-sharing and enables an open dialogue for patient questioning. Similarly, the knowledge of Lucie’s IBD nurse facilitated reassurance in her decision-making regarding treatment:

They were very knowledgeable which really helped put my mind at ease and it was the right decision for my treatment (Lucie, aged 31, CD)

Whilst knowledge emerged as a communication facilitator, six participants highlighted disparities of knowledge across the wider IBD medical community. This was apparent during an interview with one participant, who had experienced insufficient knowledge when discussing her J-Pouch with medical professionals (when the colon and rectum is removed and the end of the small intestine is used to form an internal pouch shaped like a J) (Lamb et al., 2019).

If you said J-Pouch to a medical professional, a doctor, a nurse, they go sorry what’s that?…there’s no J-pouch nurse…that stoma nurse has no idea how to deal with a J-Pouch (Natalie, aged 37, UC)

Natalie’s experience highlights the importance of specific-IBD knowledge of surgical interventions, which was perceived as creating a barrier to communicating with her HCPs. The lack of specific IBD-knowledge of dieticians was discussed by another participant:

I think certainly on the dietician side, they know their element, but they need to be more specific to the actual illness itself (Oddbods, aged 39, CD)

Perceived knowledge of various IBD professionals appeared to facilitate an open dialogue of patient-provider questioning and facilitated decision-making. However, better personalised IBD-specific knowledge such as knowledge of J-Pouch surgery, and dietary needs of individuals was emphasised by participants to facilitate communication. These findings suggest IBD-specific knowledge across mulitdisciplinary HCPs is imperative for communicating with patients in order to adequately support all individual health and healthcare related questions and concerns for people living with IBD.

#### Subtheme: Dismissing spouses

All four interviews with spouses reflected upon the integral role of the family whereby partners inevitably “talk to one another” (Stewart, aged 56, UC). However, dismissive attitudes and behaviours of HCPs towards spouses were perceived as a barrier that led to a lack of opportunity to talk with providers, share information, and be a part of the decision-making:

I’ve been there and never really been asked anything…it feels like kind of one-way traffic with the information kind of thing… I’ve never really been involved or even discussed with really...they just don’t really give you the opportunity to ask do they. (Michael, spouse)

From Michael’s experience, he discussed the need for a more collaborative and open forum of dialogue with the consultant leading his partner’s care. Interestingly, active and responsive listening skills of HCPS are previously highlighted in our study findings, and coincide with Michael’s experience. A similar experience was also elucidated by another participant.

If I chipped in ever with a comment it would be dismissed and, you know what’s it got to do with you? And thank you very much but we don’t need your input. Erm, continuously, a real kind of lack of interest in the person that can probably give you valuable information. (‘Sarah’, spouse)

It appeared the dismissive attitude of Sarah’s consultant resulted in the reduced opportunity to voice “valuable information” regarding her partners’ IBD. Sarah later emphasises how providers are “missing valuable information by not asking”. Thus, limiting abilities to share important clinical information.

Contrastingly, whilst David conceptualised that HCPs “tend to ignore spouses as a rule”, it appeared his reduced involvement was reassured by the confidence he attains in Marcia’s (CD, aged 61) providers.

I’m happy to sit in the background…it’s having the confidence in them and if Marcia’s confident in them and the treatment she’s getting I’m perfectly happy (‘David’, spouse)

Our findings suggest that lack of involvement, interaction and dismissal of spouses may potentially inhibit important clinical information being shared between spouses and HCPs. However, it appeared that confidence and reassurance from professionals contribute to a better trusting relationship between spouses and HCPs.

### Theme 2: Unequal power

The perceived social and power dynamics between participants and their HCPs was a barrier to communication discussed across participant interviews. This theme relates to the prevailing ‘expert-patient dynamics’, and ‘ impact of perceived social inequity’.

#### Subtheme: Expert-patient dynamic

Ten participants conversed about their perceptions of an expert-patient dynamic that exists between IBD patients, family members and their HCPs. One participant spoke of how the power dynamic acted as a barrier against open communication with his GP.

GPs and stuff I guess have the mentality of I’m the expert, you’re the patient, like I’m telling you what to do, this isn’t an open discussion (Saul, aged 27, UC)

This extract exemplifies how power dynamics can hinder an open discussion between patients’ and GPs involved in IBD care. From Saul’s perception, effective communication was one that is “not hierarchical.” Likewise, another participant also elucidated how power dynamics appear to influence the behaviour of HCPs:

I do find that they will speak to you almost like you’re stupid and when they do this, turn the screen away from you (Jane, aged 47, CD)

Turning the computer screen was a behaviour that signifies the power differences between Jane and her gastroenterologist. Therefore, demonstrating the importance of body language behaviours as highlighted previously in the present study findings. The importance of an equal partnership between patients and HCPs was discussed with another participant, who defined good communication as “It looks like a partnership of equals.” (Andrew, aged 62, CD).

Existing expert-patient dynamics between IBD patients, spouses and their HCPs creates a social barrier to communication. Our findings highlight how social influences inhibits open discussion, and is perceived to shape HCP body language behaviours.

#### Subtheme: Impact of perceived social inequity

Five participants clarified upon the “uneven [relationship]” (‘James’, aged 50, CD) that exists; resulting from differences in perceived income status between the person living with IBD and the HCP. One participant identified herself of being from a lower income background and explained how she felt this impacted her HCP s’ ability to listen to the difficulties she was experiencing, consequentially prolonging her diagnosis:

It was always put down to constipation erm probably being from like a poor background and always essentially fobbed off (Minnie, aged 35, UP)

Minnie’s experience highlights how from the patient perspective, coming from a low-income background was perceived a barrier to communication with HCPs. Thus, highlighting the importance of recognising how socioeconomic disparities across healthcare provision for IBD patients exists within peoples’ experiences. Intertwined with this, patient image also emerged as a barrier to feeling listened to by HCPs. One family member expressed how her partner feels they must present themselves in a formal manner to their IBD consultant and later justified.

Because you’re absolutely observed by the way you’re treated, according to what you wear and the way you present yourself (Sarah, spouse)

In the above quote, Sarah explains the importance of image in accordance with the corresponding professional attitude towards patients. This theme represents a social barrier to communication grounded within a perceived inbalance in social status between IBD patients and profiessionals which negatively impacted relationships and communication between patients and their HCPs.

### Theme 3: Patient navigation skills

When newly diagnosed with IBD, knowledge and capability to navigate through a new health care context was found to be a barrier to communicating with HCPs. Lack of ‘patient knowledge’ and ‘role uncertainty’ both emerged as barriers to communication at the earlier stages. In response, ‘tailored information resources’ were identified as a facilitator to patient knowledge, navigation and subsequent communication with their HCPs.

#### Subtheme: Patient knowledge

Eleven participants discussed they often lacked clarity as to what knowledge and questions was required of them when interacting with new IBD HCPs. Thus, consequentially impacting their confidence in communicating with their professionals:

I didn’t know what questions to ask or what knowledge I was supposed to have. You don’t know what knowledge you should have therefore you don’t know if you’re being provided with all that information (Sophie, aged 48, UC)

Sophie’s earlier experiences suggest that a lack of knowledge impacted her ability to be certain that her information needs were being met. The importance of patient knowledge was highlighted in another interview. Roy had been experiencing fistulas for two-to-three years before discovering they were a symptom of Crohn’s Disease.

If I had known it was Crohn’s, I would have probably mentioned it. I never mentioned it you see when I went to the check-up, because I didn’t know it was Crohn’s (Roy, aged 45, CD)

Roy’s experience highlights the importance of patients’ acquiring the information and knowledge they need to effectively communicate with their HCPs. This subtheme represents how patient knowledge impacts their ability to navigate and communicate through a new healthcare system.

#### Subtheme: Role uncertainty

Coinciding with this, uncertainty surrounding patients’ role and responsibility when navigating within IBD health services emerged as a barrier. One participant hadn’t seen their consultant for 10 years, and was uncertain as to their role within this process:

Maybe I don’t push enough I don’t know? Is it my job to do that? To say where’s my consultant? (Minnie, aged 35, UP)

It appeared Minnie was unsure of her role in whether she should be asking to see the consultant. In addition, one participant highlighted the difficulties individuals with IBD experience regarding accessing support.

There are people out there who want further information and want further support but just don’t know where to turn (Oddbods, aged 39, CD)

Whilst acquiring information to manage their IBD is a primary need of patients, knowledge and awareness of where and how to retrieve that imposes as a barrier to communication. Our findings highlight how people newly diagnosed with IBD require tailored resources to facilitate knowledge, and navigate their care.

#### Subtheme: Tailored information resources

The provision of information resources was found to be a facilitator to communication and patient navigation. Twelve participants discussed the use of leaflets, telephone lines and email, sharing research, and utilising secondary care IBD-specific notice boards. Providing fact sheets were shown to facilitate shared decision-making with Hector when his consultant have suggested different medication:

They’ve given me a fact sheet; I want you to have a read of this. If you have any questions come back to me…so you feel involved in the decision making (Hector, aged 57, CD)

The use of fact sheets facilitated Hector’s sense of autonomy and ownership of his treatment. Thus, facilitating patient questioning, collaboration and shared decision-making, which are important behaviours identified in this study. However, one participant offered a divergent perspective from an experience prior to an emergency colectomy. From Natalie’s perspective, her needs as a young, working-class woman were not met by the leaflet she received.

That pamphlet was not representative of somebody my age and of my concerns… so they need to be customised to dealing with that situation (Natalie, aged 37, UC)

Natalie’s experience suggests the importance of tailoring information resources to the individual. Another participant also discussed the use of research papers in facilitating communication with their HCP:

When they’ve talked about azathioprine recently and I explained like I don’t want to take it because XYZ, erm they’ve told me about new research that I didn’t even know about (Saul, aged 27, UC)

Incorporating research to support decision-making regarding treatment was an important factor for Saul when interacting with his HCPs. Furthermore, participants discussed their views of the use of notice boards within secondary care practices to facilitate patient knowledge and navigation with professionals.

…a lot of notice boards in hospitals now with information, signposting for where you can get more information...but I guess you’ve still got to keep your eyes open to see it (Marcia, aged 61, CD)

Whilst Marcia highlights hospital notice boards that can facilitate communication for patients, the need to “keep your eyes open” suggests a limited availability. Our findings suggest the appropriate provision of external information resources can act as a facilitator for shared decision-making, patient knowledge and ability to navigate to appropriate IBD services and support.

### Theme 4: Time constraints and demand

Eighteen participants voiced barriers to communication of limited time, resources and subsequent demand of their HCPs. This was perceived by participants to ‘inhibit listening skills’ and informed ‘decision-making’ between participants and their professionals.

#### Subtheme: Inhibiting listening skills

Lack of time was perceived to narrow their HCP’s ability to listen to, and support the participants individual needs and discuss concerns regarding treatment options and management of their IBD. One participant elucidated the importance of time in feeling listened to by HCPs.

Lack of time is key. Nobody seems to have time to listen to you, and to voice your concerns (Natalie, aged 37, UC)

Natalie highlights the barriers of time that limits HCPs abilities to listen to meet the concern needs of individuals with IBD. Furthermore, another participant suggested the need for more “Maybe more time for further questioning” (Lucie, aged 31, CD).

This subtheme highlights how reduced appointment timings and subsequent limited interaction with IBD HCPs inhibits communication by restricting skills of questioning and actively listening to patients.

#### Subtheme: Decision making

Participants discussed the importance of time for facilitating decision making. For Elizabeth, lack of time with patients was viewed as a barrier to well informed decision making of her HCPs:

Every health care professional I know does not have enough time to spend with their patients…the time needed to make good decisions (Elizabeth, aged 40, CD)

Elizabeth’s experience highlights how preconceptions of limited time with providers inhibits patients’ confidence in provider decision-making. Preconceptions concerning time restrictions was elaborated further when participants spoke of time pressures, and burnout of their providers; describing their HCPs as “overworked and under pressure” (Michael, spouse).

Clinic running 40 minutes late…they’re already giving you that idea, don’t go in and start waffling because things are tight (Jane, aged 47, CD)

Our findings suggest lack of time limits the ability for in depth conversation and impacts the way patients appraise the situation when entering consultations with their HCPs.

### Theme 5: Continuity and collaboration of care

All participants emphasised continuity and collaboration of their IBD care as a facilitator to communication. Consistency of interactions especially with gastroenterologists and IBD nurses appeared to facilitate trust, and the importance of collaboration across the wider healthcare context was emphasised.

#### Subtheme: Building trust

Frequent interactions and a “collaborative process” with HCPs facilitated the patient-professional relationship and trust. For many participants, trust in their HCP was a key tenant to effective communication:

The key thing for me was that I trusted my team, my medical team. And I also I built their trust in me (Ann, aged 57, CD)

This extract demonstrates how the generation of trust between Ann and all the HCPs involved in her care was a two-way process that facilitated communication. Another participant also expressed how his interaction with the same IBD nurses facilitated trust:

I think seeing the same person really…you sort of build that trust a bit more and you don’t have to say the same thing over. That really helps with the communication. (Lisa, aged 33, CD)

In addition to building trust, contact with the same HCP facilitates familiarity and patients’ ability to form a less formal relationship with their care teams. Thus, perhaps minimising the power-dynamics previously identified as a barrier.

I’ve only ever seen two consultants…we are on first name terms because I’ve been seeing him for so long (Andrew, aged 62, CD)

#### Subtheme: Collaboration between different healthcare professionals

Along with the importance of continuity and trust building, participants also explicated the importance of collaborative relationships across members of the IBD team:

They worked well together, the GP and the consultant” (Stewart, aged 56, UC) … “They had an agreement between themselves of how it was going to work and that worked really well (Mary, spouse)

Stewart and Mary elucidated upon the positive impact of Stewart’s IBD team that communicates and agrees upon his treatment plan in collaboration with both Stewart and Mary. It appears this unity across primary and secondary care professionals facilitated the treatment process, thus facilitating communication between patients, spouses and their all the healthcare team.

## Discussion

This qualitative interview study elicited IBD patients and some spouses’ perceptions of HCP communication behaviours and skills together with the barriers and facilitators. 33 HCP behaviours were grouped into eight overarching skills of: Active and responsive listening skills, promote patient autonomy, provide timely outpatient care, offer personalised care, provide helpful self-management information, tailoring language when providing information, multidisciplinary communication and body language and social cues during consultations. There were five overarching themes encompassing 11 barriers and facilitators perceived as influencing effective communication. The findings suggest the need to promote both patient and HCP knowledge, availability of resources, foster patient-provider consultations that share equal power, balance the need for time to communicate well with the reality of time restraints, and encourage collaboration across HCPs and those living with and managing IBD, including family members.

This study found similar emphasis to existing healthcare communication frameworks. As expected, several of the behaviours highlighted by patients surrounded ‘information exchange’. These could be related to patient-professional communication within communication models that recognise the importance of questioning during medical consultations (Frederikson, 1993). They are also related to behaviour change and adjustment models which emphasise the importance of helpful, tailored information to shape health beliefs, enhance perceptions of ‘relatedness’ and ‘social support’, and promote positive adjustment and coping (Deci and Ryan, 1985; Leventhal et al., 2016; Moss-Morris, 2013). Whilst there are similarities to existing communication frameworks, it is also important for HCPs in consultation with patients to consider the context of the interaction, preferred communication style, health concerns, beliefs and values of the individual (Kwame and Petrucka, 2021). Communication styles may require additional specificity to match the style of the patient group. Non-verbal behaviours were also identified such as paraphrasing, open-ended questioning, eye contact and facilitating questions from patients and souses. These serve as reminders to the busy health professional of the importance of periodically refreshing their basic communication skills training (Moore et al., 2018), ideally including role-play, patient simulation, reflective assessments and peer feedback (Henry et al., 2013). Behavioural science frameworks, including The Behaviour Change Technique Taxonomy (Michie et al., 2013), have been applied in research to incorporate behaviour change techniques into training and other health professional change interventions, to improve practice change (Ivers et al., 2012; Michie et al., 2013; Pearson et al., 2020). Those which may appear particularly helpful for ICD HCP communication skills training *Instructions on how to perform the behaviour* (how to demonstrate positive body language behaviours, give examples of questioning, and how to present treatment risks and benefits without jargon) or *Behavioural practice/rehearsal* (regular practical sessions for HCPs to practice their IBD communication skills).

Eight barriers to communication were highlighted that included a perceived lack of knowledge of the HCP, and a dismissive attitude towards spouses. Previous research highlights the perceived competency of HCPs are central to the healthcare received from IBD patients (Lesnovska et al., 2017). Our findings emphasise the integral role of family members for establishing communication, providing information, and thereby supporting individuals with IBD. We recommend the need for a family-oriented approach in adult IBD care. The better inclusion of family member perspectives within the management of long-term conditions has been shown to promote positive behaviours of individuals with diabetes and cancer (Cheraghi et al., 2015; Crespo et al., 2016). Furthermore, researchers recommend that interventions aiming to promote outcomes in long-term conditions should integrate family members, which in turn promotes patient motivation (Rosland et al., 2012). Family-centred care within IBD adult healthcare delivery may not only facilitate communication, decision-making and support; but also promote individual motivation to effectively self-manage their IBD.

Our findings highlight an existing power divide that was perceived by participants between individuals with IBD, family members and their HCPs. The social dynamic between patients, spouses and HCPs was a perceived barrier to communication; participants spoke of a perceived expert-patient power divide and those from socio-economically deprived backgrounds experienced poorer communication. Although we did not capture social deprivation quantitively (i.e. by capturing self-reported participant post codes and assessing deprivation using Index of Multiple Deprivation) in this study and this finding is categorised based on the experienced shared verbally of those participants. Whilst this is the first study to highlight this within the context of IBD healthcare delivery, previous research has demonstrated individuals from more deprived backgrounds experience more negative interactions with their professionals, including less information-sharing and interaction styles of shared-decision making compared to patients of higher income (Willems et al., 2005). This highlights a need for IBD HCPs increased awareness of the potential contextual communicative variances and the need to better empower IBD patients.

Participants’ experiences of time constraints and lack of continuity demonstrate the environmental challenges affecting HCP listening skills, relationship building and shared decision-making; of which are central for high-quality care for patients with IBD. (Lamb et al., 2019) These findings were consistent with research of nurses, that report lack of time inhibits a strong therapeutic relationship with patients (Norouzinia et al., 2015) and of studies finding continuity of care to be associated with survival rates in primary care (Maarsingh et al., 2016). This study was not the first to highlight the importance of a hospital environment that is responsive to patient needs. The appropriate use of space, continuity of care, a supportive environment and access to information resources are important to patients within NHS trust hospitals (Douglas and Douglas, 2004).

At the same time, participants highlighted that ‘people skills’ could be demonstrated in different ways. Using skills from the Motivational Interviewing approach, a counselling-approach often applied in healthcare settings to support patients with making autonomous behaviour changes, may be particularly effective in an IBD context (Mocciaro et al., 2014), in ensuring consultations are time efficient and person-centred, focussed on what the person actually wants to know (Levensky et al., 2007; Wagoner and Kavookjian, 2017). The provision of tailored resources of formats such as leaflets, telephone lines and secondary care IBD-specific notice boards were discussed to facilitate patient knowledge and navigation so that the focus of the healthcare consultation can be on developing therapeutic relationships, trust and sharing more tailored communication. This coincides with research and published guidelines that establish IBD telephone and email services as central communication pathways for patients to access support (Correal et al., 2019; Lamb et al., 2019)and UK published guidance on IBD service delivery which states the importance of IBD patients achieving information and support across all stages (Lamb et al., 2019).

Appropriate access to information are key priorities outlined amongst the key priorities in UK clinical guideline implementation (National Insititue for Health and Care Excellence, 2019). Theoretically, in this study participants spoke of the impact of information resources on patient autonomy, and the importance of relatedness within leaflet content. Self-Determination Theory highlights satisfying individual psychological needs for wellbeing that include autonomy, relatedness and competence (Ryan and Deci, 2000). Trained HCPs that support patients’ psychological needs, has a profound effect on patient behaviour, well-being, and motivation (Ryan et al., 2007) Therefore, HCPs should orient towards Self Determination Theory, supporting autonomy and promoting individual choice by increasing individualised resources to promote decision-making and a sense of self-determination of patients and family members (Migliorini et al., 2019). Moreover, recent research also highlights the variability and quality of information resources for IBD patients. A study reviewing IBD patient websites found that current website resources fail to adequately explain how symptoms persist during remission for individuals with IBD and IBS (Huisman et al., 2024).

Finally, participants discussed the need for specialist provision of psychological and psychosocial support to be integrated within services given the psychological burden IBD brings across the life course. British IBD service delivery guidelines recommend a multidisciplinary team approach to the care and management of IBD including provision of a psychologist (Lamb et al., 2019). Health psychologists or other psychologists trained to use the biopsychosocial model are needed within primary and secondary care IBD services, to help individuals with chronic health conditions to self-manage, adjust and cope with the very normal challenges of physical health conditions (Engel, 1977) With time to explore patient beliefs, concerns and expectations in more depth, enhanced psychological provision within IBD services may also help ease healthcare communication pressures on medical (nurse/doctor) staff members and carry health economic benefits, such as reductions in hospital admissions through enhanced confidence and motivation for self-care (Jepegnanam et al., 2020).

### Strengths and limitations

This study has some limitations. First, this study focused on the patient perspectives, with fewer input from the perspective of family members; other family members’ views and views of healthcare professionals are also vital pieces of the IBD communication jigsaw which need to be explored further in future studies. Almost all participants were White British and living in the UK. One participant was White American and lived in the USA. Whilst qualitative research does not aim to be fully generalisable in the same way as quantitative research, future research recruiting a wider diversity of ethnic and cultural backgrounds of individuals will help strengthen the understanding of effective communication within IBD services. By including both patients with IBD and their spouses in the interviews, this facilitated an open discussion that created a safe space for participants to speak openly about their experiences. However, this may have inhibited some spouses to speak openly in front of each other. Given that the family member sample was considerable low (*n* = 4), limiting the robustness of conclusions made towards this population.

Taking a behavioural science approach facilitated a clear picture of helpful behaviours to be gathered and a wealth of barriers and facilitators to be uncovered. The study presents a holistic, multifaceted picture of communication with clear and implementable, theory-based recommendations for enhancing IBD policy and practice.

## Conclusion

This study produced new knowledge to IBD healthcare delivery by nuancing perceived HCP communication behaviours and skills and of the barriers and facilitators to communication with IBD HCPs. Effective communication is multifaceted, encompassing factors driven by the individual (professionals and patients), environmental and social environment. Our findings add clarity and depth to current service delivery guidelines that only state the importance of ‘clear and comprehensive communication’ between patients and HCPs across both primary (GPs) and hospital care (consultants and IBD nurses), in adding understanding of what this looks like and what prevents and enables this from the patient and family perspective. This can be used to inform future policy and practice changes to improve communication between IBD patients, family members, and HCPs, facilitating evidence-based practice.

## Supplemental Material

Supplemental Material - Healthcare professional communication behaviours, skills, barriers, and enablers: Exploring the perspectives of people living with Inflammatory Bowel Disease Supplemental Material for Healthcare professional communication behaviours, skills, barriers, and enablers: Exploring the perspectives of people living with Inflammatory Bowel Disease by Rachel L Hawkins and Eleanor Bull in Health Psychology Open

Supplemental Material - Healthcare professional communication behaviours, skills, barriers, and enablers: Exploring the perspectives of people living with inflammatory bowel disease Supplemental Material for Healthcare professional communication behaviours, skills, barriers, and enablers: Exploring the perspectives of people living with inflammatory bowel diseases by Rachel L Hawkins and Eleanor Bull in Health Psychology Open

## Data Availability

The data underlying this article are available in Open Science Framework (link: https://osf.io/c5dyg/), in the article and in its online supplemental material provided.
