# Supplementary material for: Healthcare professional communication behaviours, skills, barriers, and enablers: Exploring the perspectives of people living with Inflammatory Bowel Disease
Source: Health Psychol Open. 2024 May 22;11:20551029241257782. doi: 10.1177/20551029241257782 (PMC11145995; doi:10.1177/20551029241257782)
Supplement: Supplemental Material - Healthcare professional communication behaviours, skills, barriers, and enablers: Exploring the perspectives of people living with Inflammatory Bowel Disease [file sj-pdf-1-hpo-10.1177_20551029241257782.pdf]

Supplementary File 1: Content analysis output

Healthcare professional skill	Behaviour	Example of participant quotes
**Active and Responsive Listening Skills**	Using open-ended questions	“So actually having someone just listen, and actually take on board, what’s worrying you?” (Natalie, aged 37, UC)<br><br>“Listening and asking open questions” (Jane, aged 46, CD)
	Enabling questions from patients and spouses	“Being open to receiving questions, and repeating themselves, having to repeat themselves” (Ann, aged 57, CD)<br><br>I’d be doing research as well so that when I went in, I could ask questions so. So what’s this? And erm, and that definitely helps to have a good communication channel I think is key. It shows their attention towards you” (Hector, aged 57, CD)
	Paraphrasing information	“If they sort of paraphrase it back to you and have actually listened to a word you’ve said, I think you’d come away feeling more satisfied as well” (Jane, aged 47, CD)<br><br>“Reflects back in as much as I know they’re taking on board what I’m saying to them.” (Marcia, aged 61, CD)
	Listen to the patient and/or family	“Considering the information I give” (Marcia, aged 61, CD)<br>“Well just like I said that you know that they will allow a third party to contribute to the

	member views	conversation, full stop. You know to communicate with them full stop. You know I've had people that don't even look you in the eye, don't even acknowledge that you're in the room." (Sarah, spouse)
	Consider information provided by the patient/family member	"Talking and discussing, taking into account people's points of view" (Jane, aged 47, CD)<br><br>"All those sort of things made me you know, made me feel like oh I am worth listening to, you know doctors can listen and put those preventative things in place and it is possible" (Lisa, aged 33, CD)
	Establish consultation expectations of the patient	"I think they need to listen to patient first and foremost you know what exactly is the patient wanting to get out of it?" (Mr Oddbods, aged 39, CD)
**Promote patient autonomy**	Asking the patient to track their own progress	"It's really good for me when they ask me my ideas about where I'm at. Erm, that actually engages me, involves me in tracking my own progress so I can actually tell you my last results myself and they can quickly go to them." (Ann, aged 51, CD)
	Allow patients to make their own choices	"We have had several decision-making points where she let me make a choice" (Elizabeth, aged 40, CD)

		“He knows that I am perfectly able to manage my condition, he knows that I will take Budesonide when I need it” (Andrew, aged 62, CD)
	Encourage patients to explore different options for biologics	“normally when a different drug has been suggested in the past they’ve either given me a fact sheet on this is what it is, I want you to have a read of this. If you have any questions come back to me type approach so you feel involved in the decision making that’s being made on your behalf.” (Hector, 57, CD)<br><br>“she encouraged me to go read more about what Remicade does” (Elizabeth, 40, CD)
**Provide timely outpatient care**	Provider regular check-up opportunities	“just regular contact, just checking in. They don’t check in, they just wait for the appointment and if your appointment is 12 weeks apart or what have you, there’s nothing in between just to say, are you ok?” (Minnie, 35, UP)<br><br>“I think regular catch ups and check ins I mean like I say the help-line really helped when I was first starting out as well.” (Lucie, 31, CD)
	Timely follow-ups during a flare	“they were always available and they give me erm a number to call if I ever felt you know that I was in pain or anything like that and they would get back to me within the same working day. So that was good to know that if I ever do flare again that they’re available.” (Lucie, 31, CD)
	Timely referrals from GPs	“But the GP practice who were originally there, were very very quick to refer” (David, spouse)

	Efficient at sending forms for tests	“they’re quite on top of it they send all my bloody forms to me with the date that I need to have them back in to which is very helpful because obviously I’ve still been able to work full time so it’s a lot to remember and juggle” (Lucie, 31, CD)
**Offer personalised care**	Asking the patient what is worrying them	“So actually having someone just listen, and actually take on board, what’s worrying you?” (Natalie, 37, UC)<br><br>“by understanding, with example of the follow up letter that they understand a bit about your erm personality and your anxieties around the disease. Because the anxieties plays such a huge part of the mind games of the disease as well.” (James, aged 50, CD)
	Ask about family history or comorbidities	“I need to understand what has gone on in your life, from start to finish, what erm potentially influences from family illness, your childhood illness” (Natalie, 37, UC)
	Show interest in the persons life beyond their illness	“I say they remember me when they saw me again and said ‘how’s it going? Did you ever get to New York’? things like that which were nice, I mean I didn’t but (laugh) it was nice that they remembered. I think it definitely helps when you know you’re not just someone that has Crohn’s they actually remember you and your story which is really good, I thought anyway.” (Lucie, 31, CD)<br><br>“shall we say it become less about your life is your illness, but more about the life, your life, your illness slotted in. you know where it becomes secondary because you’re a person and you’ve got a job to do, you’ve got a social, you might have a husband or wife, you’ve got other things that actually we also want to know about” (Natalie, 37, UC)
	Ask the patient about their psychological	“There’s nothing in between just to say, are you ok? Are you managing like? None of that’s there and I think that would be a huge different because they don’t know during that time if you really

	wellbeing	feel like, you need to see somebody before then.” Minnie, aged 35, UP)<br><br>“I remember him turning to me and saying and how are you? And I nearly burst out crying because I was so overwhelmed by someone not only listening to me but even caring that I was part of the equation you know” (Sarah, spouse)
**Provide helpful self-management information**	Giving detailed information of medication	“We talked through Humira, we talked through Remicade (infliximab), and we talked through Stelara” (Elizabeth, aged 40, CD)<br><br>“I think someone who will go through a bit more with the medication rather than just saying, I’m giving you something called Budesonide.” (Louise, aged 51, UC)
	Checking understanding	“Also to perhaps promote questions, what’s your understanding of that? And erm or have you looked at this or are you going to look at this?” (Hector, aged 57, CD)<br><br>“when I say clear its got to be clear to the professional and to the patient” (Stewart, aged 56, UC)
	Provide facts backed up by research data	“What am I doing? Why we’re doing it? And here’s the evidence for the why part of it.” (Saul, aged 27, UC)<br><br>“Here’s some data showing that it works, and that it’s safe. Rather than, yeah do this it will help.” (Saul, aged 27, UC)

	Explain information using scenarios	"yeah in my case what does this mean? You know me, you know my physical details you know the medication I'm on. What does this generic form mean in my case? (Hector, 57, CD)<br><br>"how they would apply to me from her practical experience and others that she'd studied with." (Elizabeth, aged 40, CD)
	Using information handouts or visuals	"That doesn't necessarily have to be talking, it doesn't have to be pictures but it can be a combination" (Stewart, aged 56, UC)<br><br>"she had a whole set of like hand-outs and you know descriptions" (Elizabeth, aged 40, CD)
	Discussing the Risks and Benefits	"They talk about like the risks of the medication as well instead of just you know, it's going to help" (Saul, aged 27, UC)<br><br>"I had an appointment with the nurse and the consultant and they talked me through the pros and cons and like the success rate, you know things like that, and that was helpful because I was a bit against going onto at first but now I don't regret it." (Lucie, aged 31, CD)
	Signpost to support services	"They will take some sort of action you know based off what you've said" (Lisa, aged 33, CD)<br><br>"I remember her looking, like really looking at my notes and saying, [gasp] you haven't had a colonoscopy for however long please can I refer you?" (Saul, aged 27, CD)
**Tailoring Language when**	Using Layman's Terms	"Layman's terms, initially talk lay man's terms maybe. I think that's a good practice thing if they

**providing information**		do talk like that." (Roy, aged 45, CD)<br><br>"You don't want to use like the scientific terms all the time in fear of being too open and forward I guess…: I feel it would have benefited me as a child because I would have told them my symptoms easier" (Saul, aged 27, CD)
	Repeat information if needed	"well as I said communicating clearly and being open to receiving questions, and repeating themselves, having to repeat themselves." (Ann, aged 51, CD)
	Use medical terminology	"I think that the health professionals I've seen have often been scared of using too much jargon…So if the doctors said why you were taking the medication, just that you should take it, I think people would adhere to it more" (Saul, aged 27, CD)<br><br>"I can understand a lot of medical terms and I want you to talk to me on those levels." (Elizabeth, aged 40, CD)
**Multidisciplinary communication skills**	Communicate with other primary or secondary care professionals	"Communication between all of my team member so my GP, and my dietician and my colorectal surgeon and my GI, they may not all talk to each other, but I need to know that they all agree with each other." (Elizabeth, aged 40, CD)<br><br>"Communication between all departments that you're dealing with" (Louise, aged 51, UC)
	Communication with GP colleagues when	"exactly because my consultant wrote to the, or after every consultation wrote to the GP like right I've seen Mr Hector, these are blood tests he need to have." (Hector, 57, CD)

	ordering investigations or prescribing new treatments	"the GP probably should have consulted the specialist before sort of making his own mind up. I think it's because, at the time my symptoms were suggestive of just like, left sided colitis so I guess that's why he went for the endoscopy because he thought he was being like smart. Erm, but if he had done the colonoscopy it would have been like killing two birds with one stone" (Saul, 27, UC)
**Body Language and social cues during consultations**	Facing the patient or family member during the consultation	"You need someone that sort of that almost turns their chair a little bit so that they are engaging with you properly, so you know they're listening to you and taking on board what you're saying." (Marcia, aged 61, CD)<br><br>"To be seen, for them to be looking at you not just listening to you." (Hector, aged 57, CD)
	Turning of chart or computer screen	"My chart is always turned so I can see it." (Elizabeth, aged 40, CD)<br><br>"Cause he was looking at it, screened turned, and I was like well can we see them please? You know, I'm really interested in what it looks like." (Michael, spouse)
	Using eye contact	"I need to have good eye contact" (Sophie, aged 48, UC)<br><br>"The eye contact as well is a really important thing isn't it." (James, aged 50, CD)
	Respond empathetically when distressed	"Just that sort of empathy and you know, like they care really about you and your situation and not being compared to anybody else and not being made to feel like you're not ill enough and that sort of thing." (Lisa, aged 33, CD)

		“Someone who’s got compassion and empathy” (Elizabeth, aged 40, CD)
