# Supplementary material for: Healthcare professional communication behaviours, skills, barriers, and enablers: Exploring the perspectives of people living with Inflammatory Bowel Disease
Source: Health Psychol Open. 2024 May 22;11:20551029241257782. doi: 10.1177/20551029241257782 (PMC11145995; doi:10.1177/20551029241257782)
Supplement: Supplemental Material - Healthcare professional communication behaviours, skills, barriers, and enablers: Exploring the perspectives of people living with inflammatory bowel disease [file sj-pdf-2-hpo-10.1177_20551029241257782.pdf]

Supplementary File 2: Thematic analysis output

Theme 1: Healthcare professional knowledge and behaviour

Extract	Initial Codes	Themes/Barriers and Facilitators
"If they know about what's wrong with you, they can understand it and they see more. It makes everything smoother doesn't it?" (Jane, Crohn's Disease)<br><br>*"Thank goodness for the IBD nurses…I think they're so knowledgeable…they explain things more, and more tests and what the medication does. They know so much and they're just more prepared to answer questions"* (Louise, Crohn's Disease)<br><br>*"They were very knowledgeable which really helped put my mind at ease and it was the right decision for my treatment"* (Lucie, Crohn's Disease)<br><br>*"Having someone who is a professional and knows what they're doing, having a calm way about them makes a world of difference"* (Sophie, Ulcerative Colitis)<br><br>*"Just knowing about it, knowing all the million and one things that come with it"* (Minnie, Ulcerative Proctitis)	Knowing the prognosis facilitates understanding and process<br><br>Knowledge to explain, answer more questions<br><br>Knowledge facilitates patient reassurance, eases anxiety<br><br>Knowledge, calmness facilitates the healthcare context<br><br>Knowledge of the wider impact of IBD	**Perceived knowledge of the professional: Facilitator**

"The understanding isn't wide throughout the whole medical community" (Jane, Crohn's Disease)<br><br>*"I don't think they know anything about it really…I remember having GPs sat there googling stuff then reading the same stuff I'd googled before I went in there"* (Saul, Ulcerative Colitis)<br><br>*"I think certainly on the dietician side, they know their element but they need to be more specific to the actual illness itself"* (Oddbods, Crohn's Disease)<br><br>*"if you said J-Pouch to a medical professional, a doctor, a nurse, they go sorry what's that?"* (Natalie, Ulcerative Colitis)	Lack of wider-IBD knowledge<br><br>Lacking GP Knowledge, use of computer<br><br>Lack of Dietician Knowledge, lack of specific IBD-knowledge<br><br>Unawareness of J-Pouch surgery with HCPs, lack of knowledge and understanding.	**Knowledge of the provider: Barrier**
"I've been there and never really been asked anything…I've never really been involved or even discussed with really?" (Michael, spouse)<br><br>*"But they just don't really give you the opportunity to talk do they?"* (Michael, spouse)<br><br>*"If I chipped in ever with a comment it would be dismissed and, you know what's that got to do with you?"* (Sarah, spouse)	Spouses not asked, not involved in discussion<br><br>Lack of opportunity to ask<br><br>Feel dismissed, opinions disregarded	**Dismissing spouses: Barrier**

"The most important thing is, they're missing valuable information by not asking" (Sarah, spouse)<br><br>*"Health professionals tend to ignore spouses as a rule. You're just there as an inconvenience during visiting times"* (David, spouse)<br><br>*"I'd be the person that lives with this and can see the patterns, can help interpret"* (Sarah, spouse)<br><br>*"It's something we've always done together"* (Stewart, Ulcerative Colitis)<br><br>*"It's great to have someone you know, your partner someone like Sarah, ok well let's get back to the agenda"* (James, Crohn's Disease)<br><br>*We've actually been able to talk to one another and we've been able to go see the consultants or nurses and say, this is what we're thinking"* (Stewart, Ulcerative Colitis)	Missing valuable information<br><br>HCPs ignore spouses, feel inconvenience<br><br>Spouses see the patterns, can interpret<br><br>Consultations done together<br><br>Spouses can regroup conversations with HCPs<br><br>Goal setting is done together	

<u>Theme 2: Unequal power</u>

Extract	Initial Codes	Themes/Barriers and Facilitators
"GPs and stuff I guess have the mentality of I'm the expert, you're the patient, like I'm telling you what to do, this isn't an open discussion" (Saul, Ulcerative Colitis)<br><br>*"We still have this thought process with the older generation that the doctor knows best"* Marcia, Crohn's Disease)<br><br>*"I suspect a lot of people fear or experience that the doctor is somehow superior to them"* (Andrew, Crohn's Disease)<br><br>*"I do find that they will speak to you almost like you're stupid and when they do this, turn the screen away from you"* (Jane, Crohn's Disease)	Expert-patient power differences, close discussion<br><br>Doctor knows best mentality<br><br>Perceived superiority of doctors<br><br>Intelligence differences, turning of screen	**Expert-patient dynamic: Barrier**
"I'd be crying saying I'm really struggling, I'm in so much pain and they would write on letters, she looks great. Because I've gone in with hair, makeup and clothes…how should I look in order to make it clear to you I'm	Image and appearance effects HCP ability to listen	**Impact of perceived social inequity: Barrier**

having difficulties?" (Natalie, Ulcerative Colitis)		
"Because you're absolutely observed by the way your treated according to what you wear and the way you present yourself" (Sarah, spouse)	Image and appearance effects the way you are treated	
"He's just the type where if you're going in your twenties-thirties and you look alright then you must be alright kind of approach" (Oddbods, Crohn's Disease)	Young age perceived of good health	
"The relationship is always a bit uneven because…they're clearly going to be middle class you know" (James, Crohn's Disease)	Uneven relationship, status	
"It was always put down to constipation erm probably being from like a poor background and always essentially fobbed off" (Minnie, Ulcerative Proctitis)	Lower SES feel dismissed	

Theme 3: Patient navigation skills

Extract	Initial Codes	Themes/Barriers and Facilitators
"I didn't know how to represent my history accurately to her" (Elizabeth, Crohn's Disease)<br><br>*"I didn't know what questions to ask or what knowledge I was supposed to have. You don't know what knowledge you should have therefore you don't know if your being provided with all that information"* (Sophie, Ulcerative Colitis)<br><br>*"So knowing when to hand over to the experts and knowing when to, you know you need help"* (Ann, Crohn's Disease)<br><br>*"If I had known it was Crohn's, I would have probably mentioned it. I never mentioned it you see when I went to the check-up, because I didn't know it was Crohn's"* (Roy, Crohn's Disease)	Knowledge of how to present history to HCPs<br><br>Don't know what questions to ask, what knowledge is needed<br><br>Knowing when to ask for help<br><br>Lack of awareness of secondary IBD symptoms	**Patient knowledge: Barrier**
"Maybe I don't push enough I don't know? Is it my job to do that? To say where's my consultant?" (Minnie, Ulcerative Proctitis)	Unaware of role when asking to see HCPs	

"There are people out there who want further information and want further support but just don't know where to turn" (Oddbods, Crohn's Disease)<br><br>*"It's maybe more difficult newly diagnosed because they don't know people and they just have to go on GP referrals as to who they see"* (Marcia, Crohn's Disease)<br><br>*"Not only did I not know how to manage this myself anyway, but like I couldn't even ask. I felt I couldn't ask a doctor, a specialist as well like how to deal with it"* (Louise, Crohn's Disease)<br><br>*What do I do, I can't find the information, I can't find any assistance? Is there someone who can help me with diet?...pain control…provide me with some form of emotional and mental counselling?"* (Natalie, Ulcerative Colitis)	Where to find knowledge?<br><br>Newly diagnosed don't know the system<br><br>Feel as though they can't ask<br><br>Where to find the information? who to turn to for specific assistance	**Role uncertainty: Barrier**
"She gave me a big information pack which helped a lot" (Lucie, Crohn's Disease)	Information sheets to understand symptoms	**Tailored information resources: Facilitator**

"When I turned up, she had a whole set of hand-outs and you know, descriptions of research papers and she like immediately understood what I needed" (Elizabeth, Crohn's Disease)<br><br>*"They've given me a fact sheet; I want you to have a read of this. If you have any questions come back to me…so you feel involved in the decision making"* (Hector, Crohn's Disease)<br><br>*"He was very good at doing things like diagrams…[good for] explaining the extent of their procedures of the area of my gut actually effected"* (Stewart, Ulcerative Colitis)<br><br>*"For primary care…to have more signposting for patients to look into their own symptoms more, simple information sheets"* (Marcia, Crohn's Disease)<br><br>*"Crohn's and Colitis UK have an awful lot of notice boards in hospitals now with information, signposting for where you can get more information...but I guess you've still got to keep your eyes open to see it"* (Marcia, Crohn's Disease)	Hand-outs, understanding patients' needs<br><br>Fact sheets to make informed decision<br><br>Use of diagrams to explain surgical procedures<br><br>Signposting, symptom specific resources<br><br>Hospital notice board to educate, needs to be more obvious	

"And if I had questions, I was given the facility to ring my IBD nurse that I could contact" (Sophie, Ulcerative Colitis)<br><br>*"I've got access to email and contact telephone as well if I need to see them"* (Oddbods, Crohn's Disease)<br><br>*"It's open communication. I can email him now, I email my results…so you know we talk via email"* (Andrew, Crohn's Disease)	IBD telephone lines to ask questions<br><br>Email and telephone extend communication<br><br>Email opens communication	

Theme 4: Time constraints and demand

**Extract**	**Initial Codes**	**Themes/Barriers and Facilitators**
"I don't know how much time they would have to do it if I needed some emotional support" (Louise, Crohn's Disease)<br><br>*"Clinic running 40 minutes late or whatever so they're already giving you that idea, don't go in and start waffling because things are tight"* (Jane, Crohn's Disease)	Limited time for support<br><br>Pre-empting push for time, limits conversation	**Inhibiting listening skills: Barrier**

“maybe more time for further questioning” (Lucie, Crohn’s Disease)<br><br>*“Nobody seems to have time to listen to you, and to voice your concerns”* (Natalie, Ulcerative Colitis)<br><br>*“you’re barely sat down and he wanted you out the room. It was really quick”* (Louise, Ulcerative Colitis)<br><br>*“when you’ve literally got 8 minutes with a consultant I understand there’s a need to knock out the questions quickly”* (James, Crohn’s Disease)	Lack of time limits in-depth questioning<br><br>Lack of time to listen, discuss concerns.<br><br>Feeling rushed, pre-empted feeling<br><br>Acknowledgment of 8 minutes, fast paced questioning.	
“Every health care professional I know does not have enough time to spend with their patients…the time needed to make good decisions” (Elizabeth, Crohn’s Disease)<br><br>*“They have to come to a conclusion quickly based on what you’ve told them”* (Lucie, Crohn’s Disease)	Time to spend with patients, decision making<br><br>Limited time means fast decisions	**Decision-Making: Barrier**

"I don't want to bother the NHS when I know how much stress they are under" (Minnie, Ulcerative Proctitis)<br><br>*"I do sometimes get the feeling that there's time pressure"* (Andrew, Crohn's Disease)<br><br>*"They're completely overworked and they're under pressure"* (Michael, spouse)<br><br>*"…being treated like a nuisance, they're too busy, they can't do this"* (Marcia, Crohn's Disease)	Feeling a burden, stress of HCPs<br><br>Feel the time pressure<br><br>Overworked staff, pressure upon staff<br><br>Feel a nuisance, too busy to help	

<u>Theme 5: Continuity and collaboration of care</u>

**Extract**	**Initial Codes**	**Themes/Barriers and Facilitators**
"They worked well together, the GP and the consultant" (Stewart, Ulcerative Colitis). *"They had an agreement between themselves of how it was going to work and that worked really well"* (Mary, spouse)	HCPs working together in agreement	**Collaboration between professionals: Facilitator**

"I got an appointment with the consultant and a nurse, which was great" (Louise, Crohn's Disease)<br><br>*"Communication between all my team members so my GP, dietician and my colorectal surgeon and my GI"* (Elizabeth, Crohn's Disease)<br><br>*"The medical experts all sat round and had a full discussion of what was best and presented that to me"* (Oddbods, Crohn's Disease)<br><br>*"I became a person in that multidisciplinary team, rather than apart from that team…I felt I had a voice"* (Ann, Crohn's Disease)<br><br>*"The problems are as a result of the disjointedness between other departments…they don't talk together, they don't share information, you know as the bigger picture for your health"* (Natalie, Ulcerative Colitis)<br><br>*"You get one team who had a different culture to another team"* (Sarah, spouse)<br><br>*"So a massive barrier is the lack of data sharing, record collection and general*	Joint consultant and nurse appointment<br><br>Communication across IBD team<br><br>Discussion and inclusion of patient in the decision<br><br>Apart of the MDT, voice<br><br>Disjointedness of departments, lack of info sharing<br><br>Staff culture<br><br>Lack of data sharing, records, communication across areas	

information and communication across different areas of the NHS" (Sarah, spouse)		
"I had the same nurses for a year…we built up a good relationship with her coming to see me every eight weeks" (Lucie, Crohn's Disease)<br><br>*"I've seen the same person for ten years, so she knows, she knows me inside out and the family as well"* (James, Crohn's Disease)<br><br>*"By then to have always known who I am, known me, instantly they don't need me to give them a refresher…it kind of saves a lot of time when you don't have to go over things"* (Minnie, Ulcerative Proctitis)<br><br>*"I always see my consultant fortunately…It's a huge barrier if you don't have the continuity"* (Marcia, Crohn's Disease)<br><br>*"It's the same person seeing me each time which is really good because you sort of, feel like your building a rapport and they understand what you're going through"* (Lisa, Crohn's Disease)	Same nurses builds relationship<br><br>Same person knows the patient and family<br><br>Knows the person, no need to go over history, saves time<br><br>Regular contact<br><br>Builds rapport	**Building trust: Facilitator**

"Since I've had the IBD nurse, I see one of three. And I've got to know them and trust them" (Roy, Crohn's Disease)<br><br>*"Cause I've only ever seen two consultants…we are on first name terms because I've been seeing him for so long"* (Andrew, Crohn's Disease)<br><br>*"And the key thing for me was that I trusted my team, my medical team. And I also I built their trust in me"* (Ann, Crohn's Disease)	Builds trust in HCPs<br><br>Less formal interactions.<br><br>Building trust with one-another.	
